# Inhibition of neuronal autophagy mediates longevity

**DOI:** 10.18632/aging.101297

**Published:** 2017-09-30

**Authors:** Jonathan Byrne, Thomas Wilhelm, Holger Richly

**Affiliations:** Laboratory of Molecular Epigenetics, Institute of Molecular Biology (IMB), 55099 Mainz, Germany

**Keywords:** aging, antagonistic pleiotropy, autophagy, C. elegans, neurodegeneration

Aging describes the age-dependent decline in the physiological functions of an organism, which affects fitness and predisposes for age-related diseases. A prominent feature of aging is the impairment of cellular homeostasis, which contributes to the emergence of neurodegenerative diseases such as Alzheimer's, Parkinson's and Huntington's disease [1]. One critical process that maintains cellular metabolism and homeostasis is macroautophagy (referred here to as autophagy). This highly conserved bulk degradation program engulfs dysfunctional organelles and misfolded proteins by double membrane structures to form autophagosomes. The subsequent fusion of autophago-somes with lysosomes leads to the degradation and recycling of the internalized content. Autophagy exists at basal levels for constitutive turnover of cytosolic components, whereas elevated autophagic levels allow the organism to adapt to adverse physiological conditions such as nutrient deprivation. Functional autophagy is generally believed to counteract aging and is essential for the prolonged lifespan observed in the context of reduced insulin- or TOR-signaling [2]. Surprisingly, and in stark contrast to its longevity promoting effects, we have recently demonstrated that post-reproductive inhibition of the autophagic nucleation complex strongly extends *C. elegans* lifespan and contributes to improved neural integrity. Our study shows that these opposing effects of autophagy on lifespan agree with the predictions of the antagonistic pleiotropy hypothesis of aging, which was formulated 60 years ago by George C. Williams [3]. This evolutionary based hypothesis predicts that some genes mediate beneficial effects early in life when natural selection is strong, but are detrimental late in life when natural selection is weak. Genes with pro-fitness and anti-longevity effects can be actively selected for and accumulate over generations, driving the aging process forward

To the best of our knowledge, we performed the first late-life RNAi screen designed to discover novel longevity genes that exhibit antagonistic pleiotropy in *C.elegans* [4]. Through this novel screening approach, we found that post-reproductive inactivation of the prominent forkhead box (FOX) A transcription factor PHA-4 results in longevity, while its inactivation in early life shortens lifespan. Previous studies have shown that PHA-4 governs the expression of autophagy genes and thereby mediates longevity in diet-restricted and germline-less animals. This prompted us to investigate if autophagy specific genes also show antagonistic pleiotropy and extend *C. elegans* lifespan when inactivated after reproduction. We discovered that post-reproductive inactivation of genes required for the autophagosome nucleation, such as the Atg6/VPS30/beclin 1 ortholog *bec-1*, led to a strong lifespan increase of up to 60% post-RNAi initiation. Just like *pha-4*, these autophagy genes reduced lifespan when inactivated early in life, which is in line with previous observations [5]. Interestingly, post-reproductive inactivation genes that control later steps of the autophagic cascade (e.g. vesicle elongation, maturation, and cargo degradation) did not positively affect *C. elegans* lifespan. We could further show that, while the process of vesicle nucleation is still active and possibly enhanced in old worms, the process is blocked downstream of autophagosome biogenesis at the step of autolysosomal degradation [4]. We moreover found that post-reproductive inactivation of autophagosome nucleation extends lifespan primarily through the neurons. This is surprising, as neurodegenerative diseases are generally characterized by the toxic accumulation of protein aggregates [6], which should normally be targeted by and degraded through autophagy. Correspondingly, it has been shown that the suppression of basal autophagy causes neurode-generation in mouse neurons. Further, autophagic stimulation has been linked to neuroprotective phenotypes [7]. At first glance, our findings seem at odds with these reports. There is, however, no contradiction when we take into account the different time frames been considered. The aforementioned studies show that neurodegeneration arises as a consequence of disrupting autophagy in young animals. In older animals, autophagy is already dysfunctional and actively contributing to a decrease in neural integrity. Thus, by bypassing the system in its early stages we alleviate the toxic effects of dysfunctional autophagy on the neurons increasing neural integrity and consequently lifespan [4].

The post-reproductive inhibition of vesicle nucleation not only resulted in increased longevity but also increases *C. elegans* healthspan. This improvement in health was evidenced through an enhanced muscle function and integrity upon global or neuron-specific inactivation of *bec-1* after reproduction [4]. On the other hand, inactivation of vesicle nucleation specifically in muscle tissues did not ameliorate age-related sarcopenia. Thus, it is specifically the effect on the neurons alone which translates into improved muscle health. As post-reproductive inactivation of *bec-1* also improved neuronal health [4], it is likely that the delay in sarcopenia is due to an enhanced muscle innervation at old ages. However, the action of additional cell non-autonomous signals from the neurons cannot be ruled out.

The molecular mechanism underlying the observed neuroprotection following post-reproductive *bec-1* inactivation still needs to be unveiled. Our study shows an accumulation of dysfunctional autophagosomes through ongoing vesicle nucleation but impaired autolysosomal degradation at old age. Interestingly, such a phenotype has often been described in the neurons of patients with Alzheimer's, Parkinson's, and Huntington's disease. Further, animal and cell culture studies of neurodegenerative diseases suggest that accumulating autophagosome along the microtubule network leads to a collapse of the axonal retrograde transport and causes neurodegeneration. While further research is still needed to characterize the status of the autophagic flux in aged *C. elegans* neurons, our study hints that the global dysfunction of autophagy observed also occurs in the neurons and results in shortened life. From a therapeutic perspective, the autophagic vesicle nucleation complex may prove to be a good pharma-cological target in the treatment of neurodegenerative diseases.
